# Primary extranodal soft-tissue B-cell lymphoma with abundant immunoglobulin inclusions mimicking adult rhabdomyoma: a case report

**DOI:** 10.1186/1752-1947-5-53

**Published:** 2011-02-07

**Authors:** Zeng-Shan Li, Pei-Feng Li, Zhe Wang, Gao-Sheng Huang

**Affiliations:** 1 Department of Pathology, State Key Laboratory of Tumor Biology, Xi-Jing Hospital, Xi'an, Shaanxi 710032, Peoples Republic of China

## Abstract

**Introduction:**

Immunoglobulin inclusions are found in B-cell neoplasms as well as in crystal-storing histiocytosis associated with B-cell lymphoproliferative disorders. At times, the deposits may be so profound as to obscure the diagnosis and may even lead to misdiagnosis. We report one case of low-grade extranodal lymphoplasmacytic lymphoma with abundant immunoglobulin inclusions and emphasize the need for immunophenotyping and molecular assay to make the right decision in diagnosis. To the best of our knowledge, this is the first report of extranodal B-cell lymphoma with abundant intracellular immunoglobulin accumulation.

**Case presentation:**

A 62-year-old Asian man from China presented with a 13-year history of a right shoulder mass with recent ongoing pain. A desmoplastic fibroma located in the posterior muscles of the neck was suggested by magnetic resonance imaging, and extended local excision was performed. A biopsy, however, revealed large, isolated rhabdoid cells in a diffuse pattern with mild atypia and eosinophilic cytoplasm. Clustered lymphoid cells were interspersed among these cells. The diagnosis was initially suggested to be adult rhabdomyoma. The final diagnosis of lymphoma was made after immunohistochemical, ultrastructural and molecular studies.

**Conclusion:**

We emphasize this histopathologic and immunohistochemical finding because of the potential for confusion with other tumors or disorders, such as adult rhabdomyoma or crystal-storing histiocytosis.

## Introduction

Well-developed immunoglobulin (Ig) inclusions, such as Russell or Dutcher bodies, are very important diagnostic clues for some B-cell lymphomas, but diffuse and abundant intracellular Ig inclusions are unusual. Another condition in which unusual intracytoplasmic Ig inclusions can be found is crystal-storing histiocytosis associated with B-cell lymphoproliferative disorders. All of these factors may lead to a diagnostic dilemma [1,2]. We describe one case of low-grade lymphoplasmacytic lymphoma with abundant Ig inclusions and emphasize the need for the use of immunophenotyping and molecular assays to make the right decision in diagnosis and to distinguish these unusual cases from those involving other tumors or disorders.

## Case presentation

**Figure 1:**
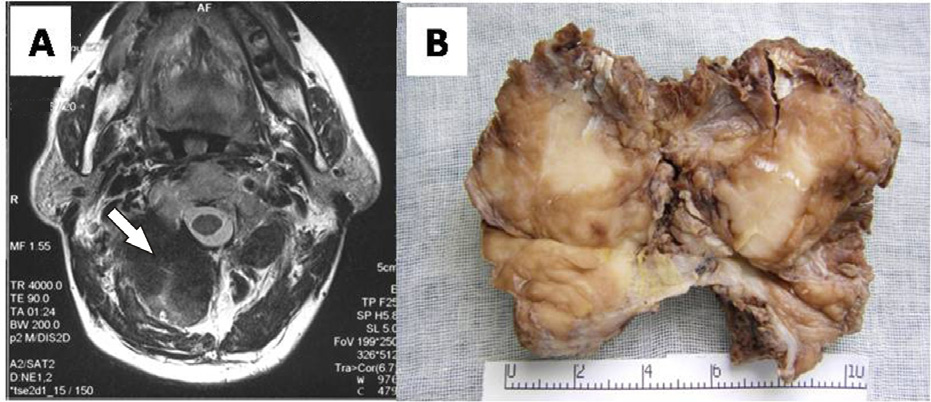
**Views of the tissue mass**. (a) Magnetic resonance imaging scan. A soft-tissue mass is seen in the right shoulder (white arrow). (b) Gross morphology.

A 62-year-old Asian man from China with a 13-year history of an enlarging right shoulder mass and recent ongoing pain was admitted to our hospital. His cervical lymph nodes were not palpable, and a review of all other systems was negative. A chest radiograph was normal. A desmoplastic fibroma located in the posterior muscles of the neck was suspected on the basis of computed tomography (CT) and magnetic resonance imaging (MRI) (Figure 1a), and extended local excision was performed. An 8 cm × 6 cm poorly defined, intramuscular, homogeneous solid mass (Figure 1b) was studied with histology, histochemistry, immunohistochemistry, ultrastructural studies and molecular genetics.

**Figure 2:**
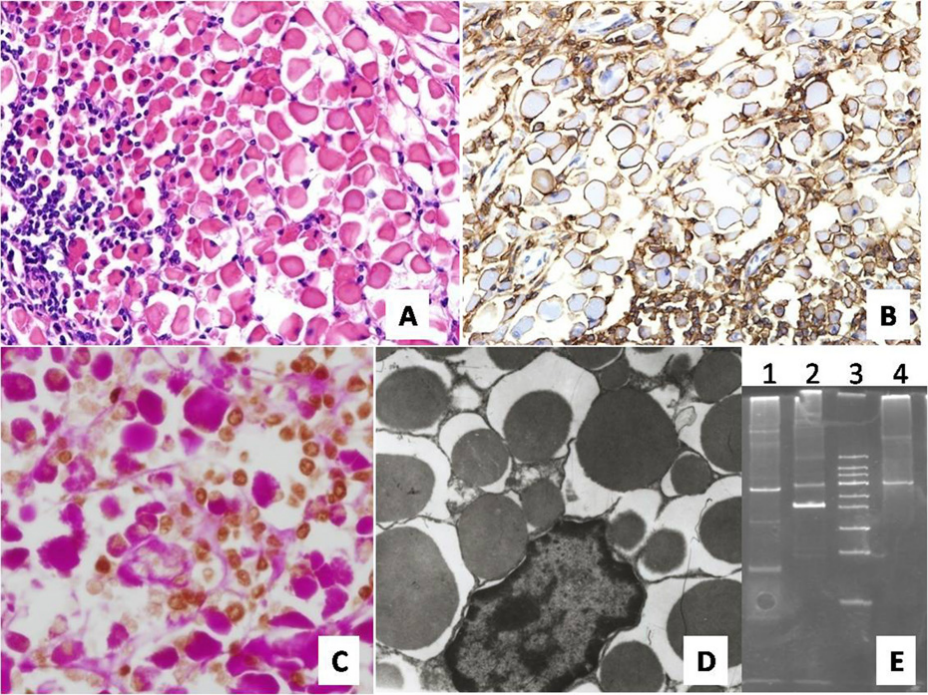
**Results of studies**. (a) Hematoxylin and eosin staining (original magnification, ×400). (b) Immunohistochemical detection of CD79α (original magnification, ×400). (c) Double staining with periodic acid-Schiff stain (histochemistry) and paired box protein 5 (PAX5) (immunohistochemistry). (d) Electron microscopic micrograph of inclusion (original magnification, ×7,250). (e) Lane 1 is immunoglobulin heavy chain (IGH) rearrangements, lane 4 is immunoglobulin light chain kappa (IGK) rearrangements and lane 3 is a 50bp ladder.

Characteristically, the excised tumor was composed of large, isolated rhabdoid cells in a diffuse pattern with mild atypia and eosinophilic cytoplasm (Figure 2a). These cells were interspersed with clustered lymphoid cells. The diagnosis was initially suspected to be adult rhabdomyoma, but the tumor cells were negative for immunostaining of desmin, muscle actin (HHF35), α-smooth muscle actin (α-SMA) and myoglobin and positive for periodic acid-Schiff with diastase (PAS-D). Then a broad spectrum of immunohistochemical stains were used, and the tumor cells were immunoreactive for CD79α (Figure 2b), CD20, CD43, paired box protein 5 (PAX5), leukocyte common antigen (LCA), and partially for CD5 (Additional file 1), but negative for CD138, CD68, CD163, CD3, synaptophysin and neuron-specific enolase. Ki-67 positivity was unevenly distributed in different areas, and the incidence was greater in the clustered lymphoid cells and almost zero in adjacent rhabdoid cell areas (Additional file 1). More interestingly, some of the eosinophilic inclusions were located in the nucleus, as demonstrated by periodic acid-Schiff stain (PAS)-PAX5 double staining (Figure 2c). Electron microscopy revealed pools of homogeneous, spherical, electron-dense bodies located in significantly dilated rough endoplasmic reticulum as well as extracellularly (Figure 2d). The mitochondria and lysosomes were poorly developed. Furthermore, monoclonality of the tumor cells was demonstrated by the presence of immunoglobulin heavy chain (IGH)-specific and immunoglobulin light chain kappa (IGK)-specific rearrangements (Figure 2e).

Other postoperative examinations showed that levels of IgE were elevated (332IU/mL) and levels of IgA, IgM and IgG were normal. Bence-Jones protein was not found in the patient's urine. The final histopathologic diagnosis was consistent with a low-grade lymphoplasmacytic lymphoma with abundant Ig inclusions.

## Discussion

Igs are reliable markers for the recognition of B lymphocytes and their neoplasms. In B-cell neoplasms, monotypic Ig is usually detected [3]. Igs may be located in the surface membrane, rough endoplasmic reticulum and perinuclear space. Well-developed Ig inclusions may appear as Russell or Dutcher bodies and give the strongest clue for the diagnosis. The Ig inclusions may be found in a range of B-cell neoplasms, including chronic lymphocytic leukemia, marginal zone B-cell lymphoma and follicular center cell lymphoma [4]. These inclusions are usually large, loosely packed fibrillar material not surrounded by a membrane or by rough endoplasmic reticulum, rodlike crystalline structures and, more rarely, signet ring-like vacuoles filled with microvesicles [5]. At times, the deposits may be so profound as to obscure the diagnosis and may even lead to misdiagnosis. In addition to lymphoma, unusual intracytoplasmic Ig inclusions can be found in crystal-storing histiocytosis associated with B-cell lymphoproliferative disorders such as diffuse large B-cell lymphoma, myeloma, lymphoplasmacytic lymphoma, marginal zone lymphoma and even non-neoplastic plasma cell proliferation [1,2]. It is a rare phenomenon in which macrophages accumulate light-chain or Ig crystalline inclusions [6]. All of these factors emphasize the need for immunophenotyping to make the right decision in diagnosis and distinguish these unusual cases from other tumors or disorders [2].

The case reported here was initially considered rhabdomyoma because of the morphologic features, the patient's age and the location of the tumor. The initial diagnosis was ruled out by immunohistochemical analysis that showed that tumor cells were negative for desmin, HHF35, α-SMA and myoglobin and positive for PAS-D. Then we checked the slides carefully and found some Russell body-like intercellular eosinophilic bodies, which have the same characteristics as those in the cytoplasm, as well as the transition from small lymphoplasmacytic cells to those unique, large rhabdoid cells. Some other associated diseases were included in the differential diagnosis, including crystal-storing histiocytosis, extramedullary myeloma, and other Ig deposition diseases [1,7]. Finally, a special low-grade lymphoplasmacytic lymphoma with abundant intracytoplasmic Ig inclusions was demonstrated on the basis of immunohistochemical, ultrastructural and molecular studies. To the best of our knowledge, this is a rare case of primary malignant lymphoma with abundant intracytoplasmic Ig inclusions, as well as Dutcher bodies. The inclusions may be an outcome of impaired cell secretory activity, leading to Igs accumulation. It also may be associated with the excess production of a monoclonal Ig. It is not unusual that B-cell-associated disorders contain intracellular or intranuclear inclusion bodies such as Russell bodies, Dutcher bodies and other crystalline structures. Most of them are minor and not so evident compared with what we found in this case.

## Conclusion

We report a rare case of low-grade B-cell lymphoma with abundant intracellular Ig accumulation. The patient's clinical presentation and MRI suggested a soft-tissue tumor, and histopathologic studies showed special rhabdoid cells with interspersed clustered lymphoid cells, thus providing a diagnostic dilemma. The final diagnosis was made with immunohistochemical, ultrastructural and molecular studies. It is important for pathologists to be aware of this rare extranodal Ig-rich lymphoma because of the potential for confusion with other tumors, such as rhabdomyoma and crystal-storing histiocytosis.

## Competing interests

The authors declare that they have no competing interests.

## Consent

Written informed consent was obtained from the patient for publication of this case report and accompanying images. A copy of the written consent is available for review by the Editor-in-Chief of the journal.

## Authors' contributions

ZSL and PFL contributed equally to this work as first authors by providing data collection, data analysis and interpretation and manuscript writing. ZW and GSH contributed to the conception and design of the report. All authors read and approved the final manuscript.

## Supplementary Material

Additional file 1 **Supplementary immunohistochemical results**. Immunohistochemical detection of LCA, PAX-5, CD5, CD20, CD43 and Ki-67 (original magnification, ×400). Click here for file
