# Supplementary figures and images for: Primary extranodal soft-tissue B-cell lymphoma with abundant immunoglobulin inclusions mimicking adult rhabdomyoma: a case report
Source: J Med Case Rep. 2011 Feb 7;5:53. doi: 10.1186/1752-1947-5-53 (PMC3041763; doi:10.1186/1752-1947-5-53)

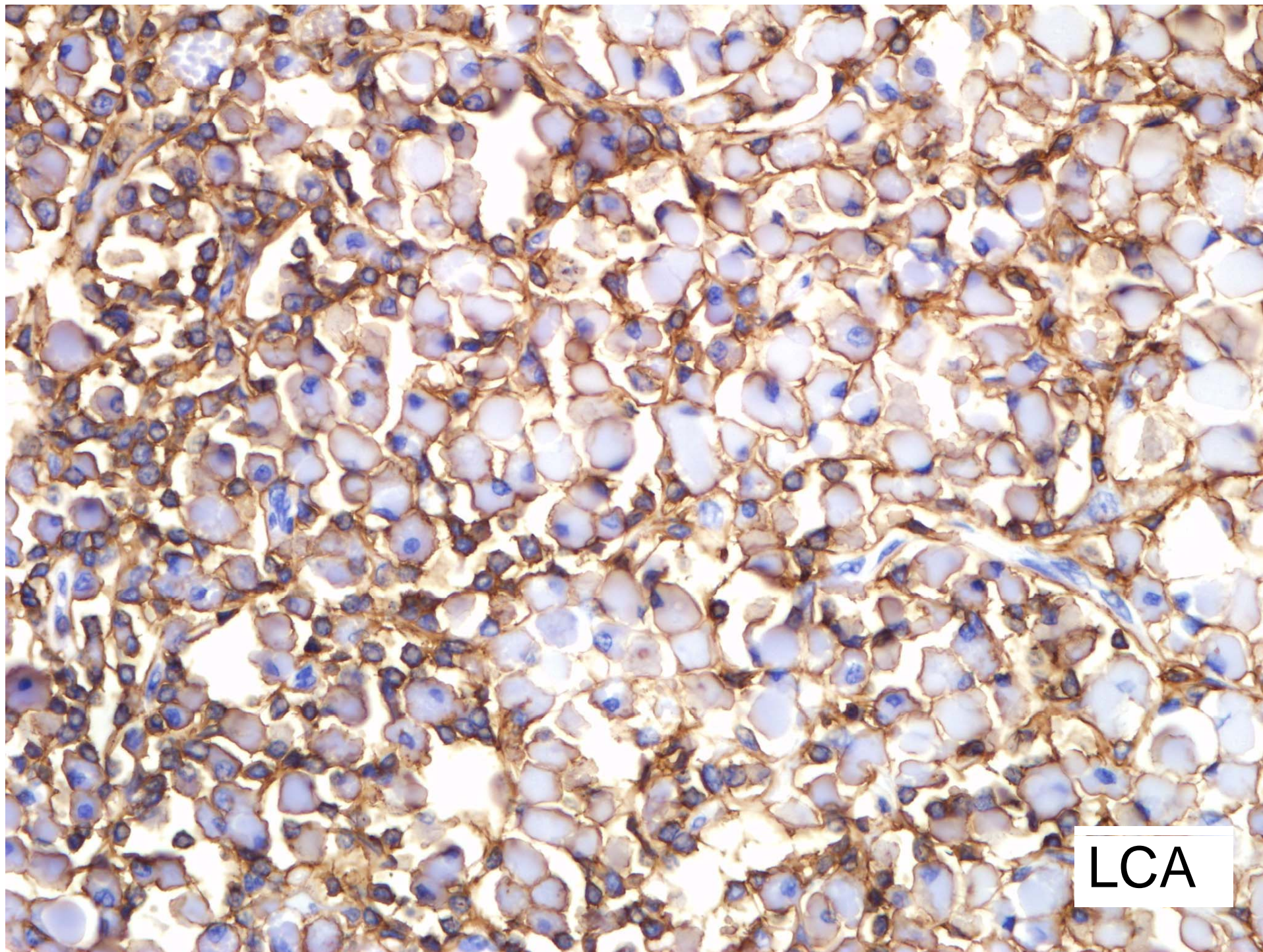

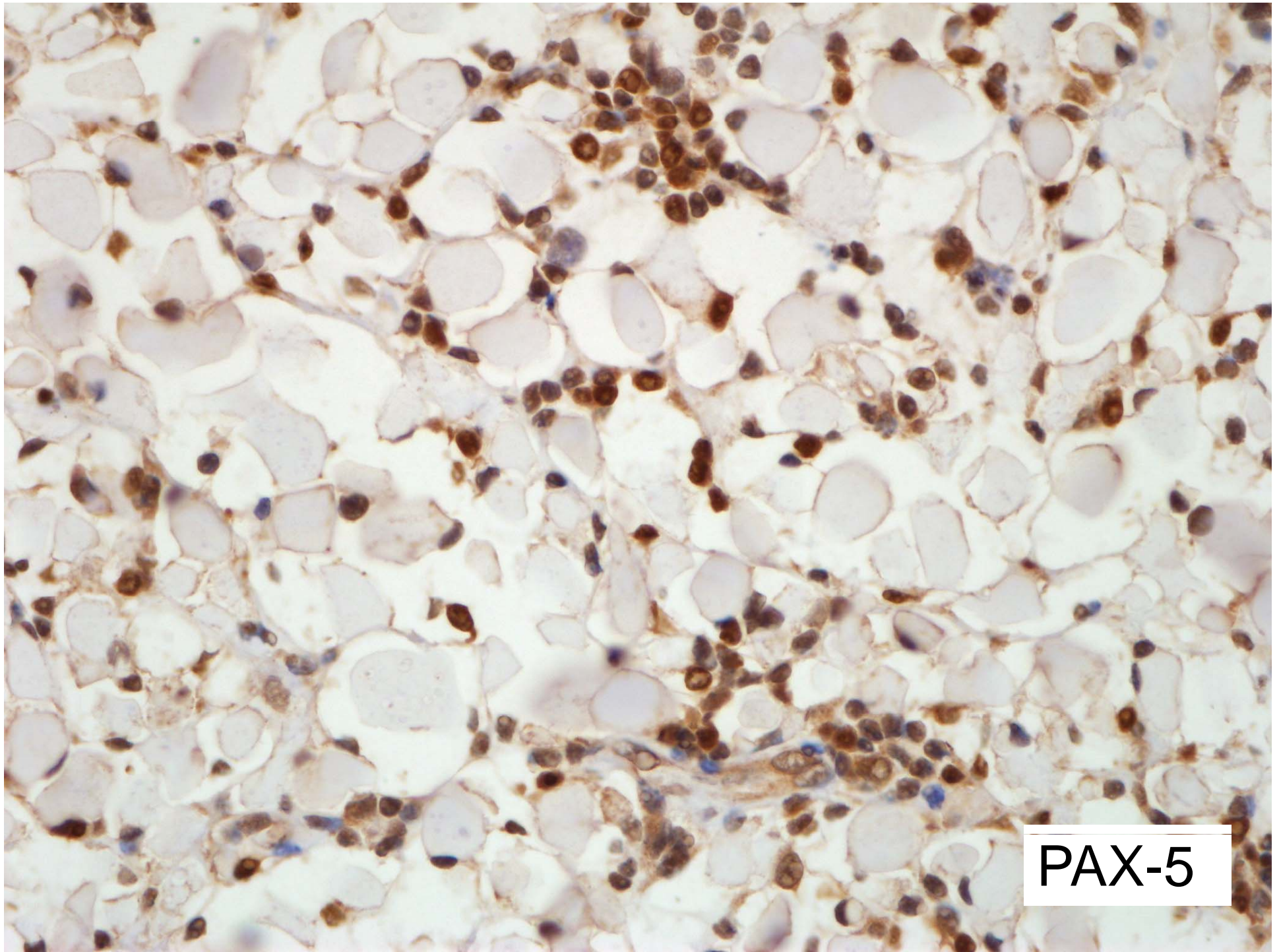

PAX-5

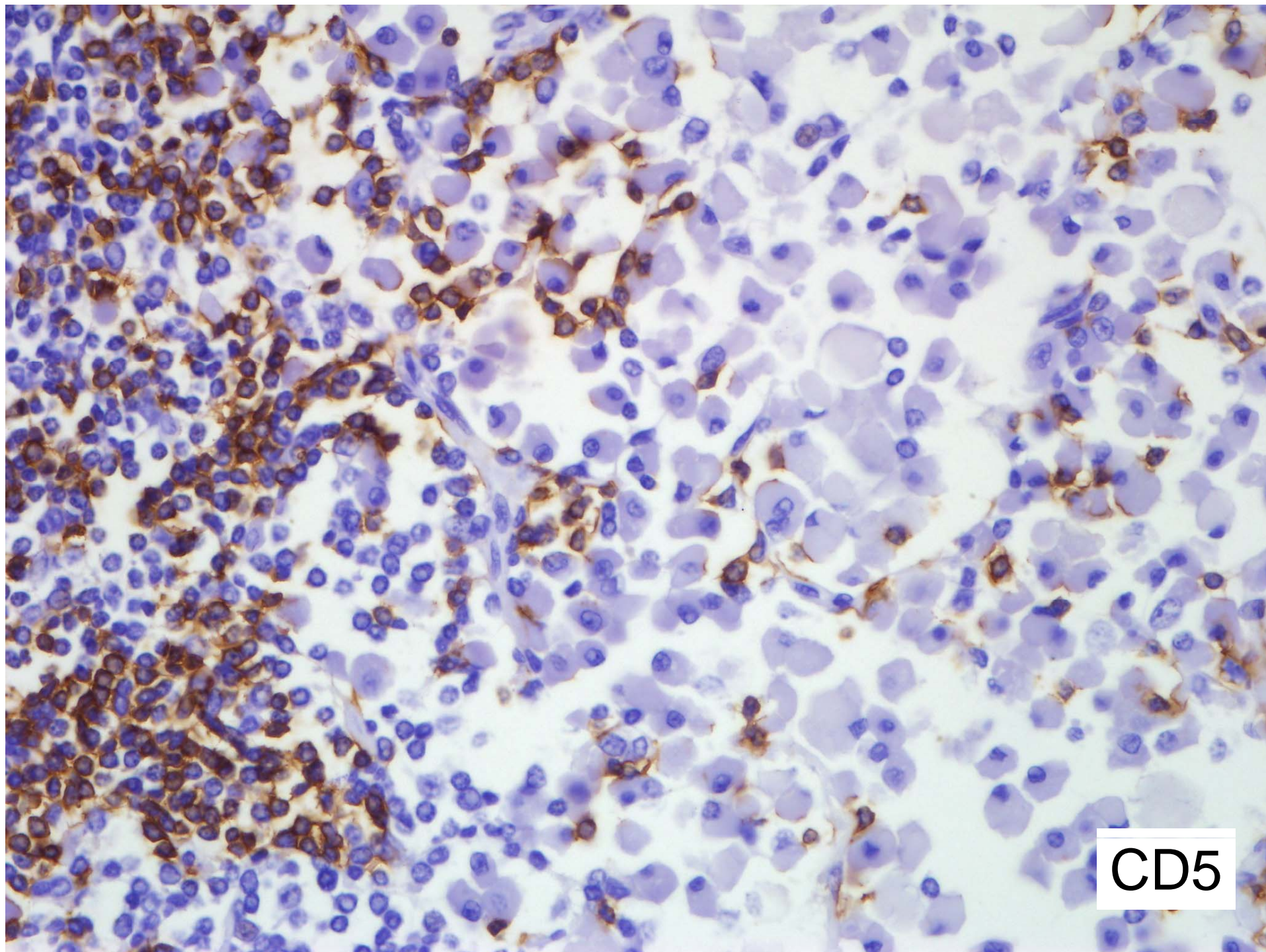

CD5

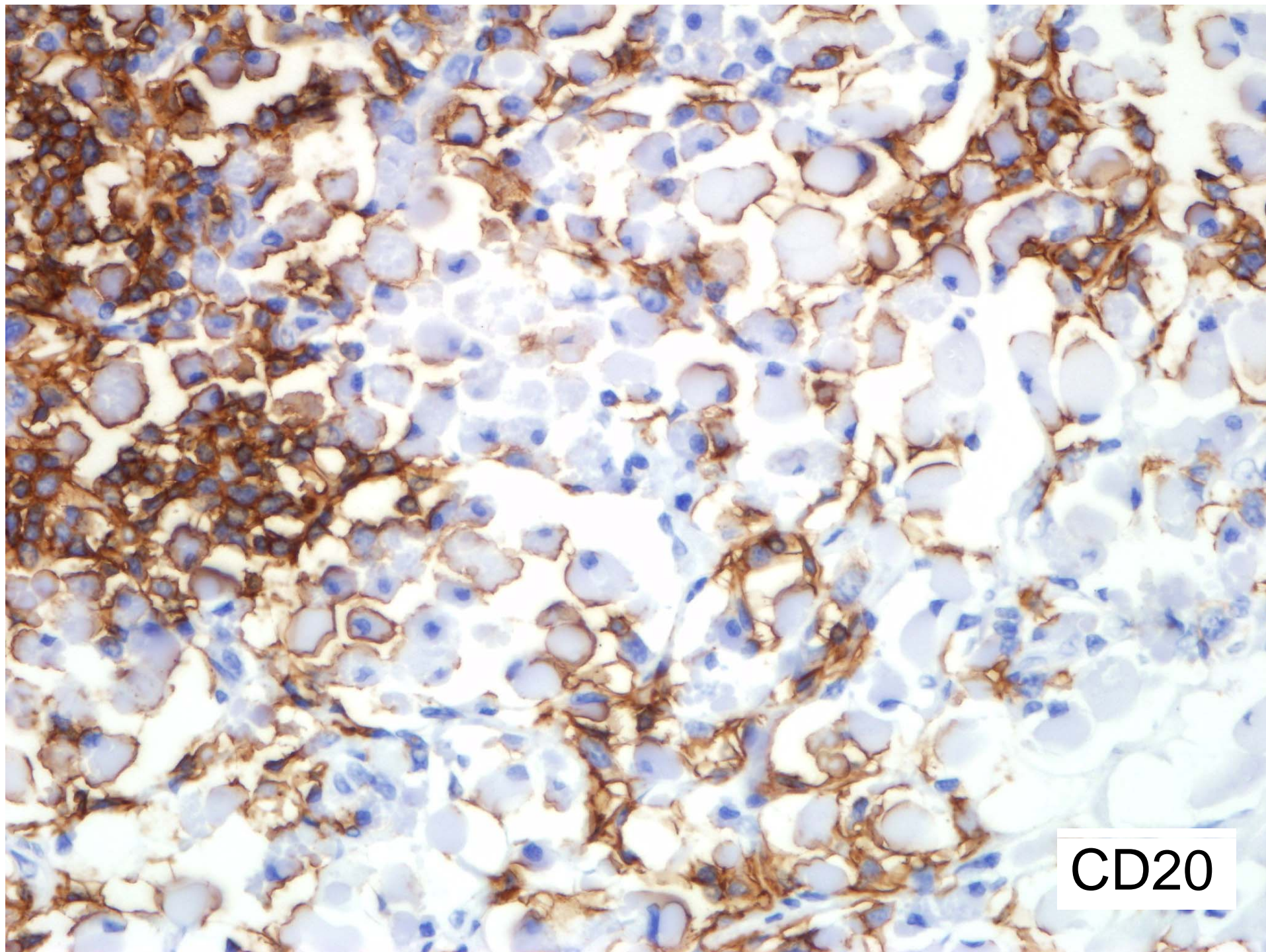

CD20

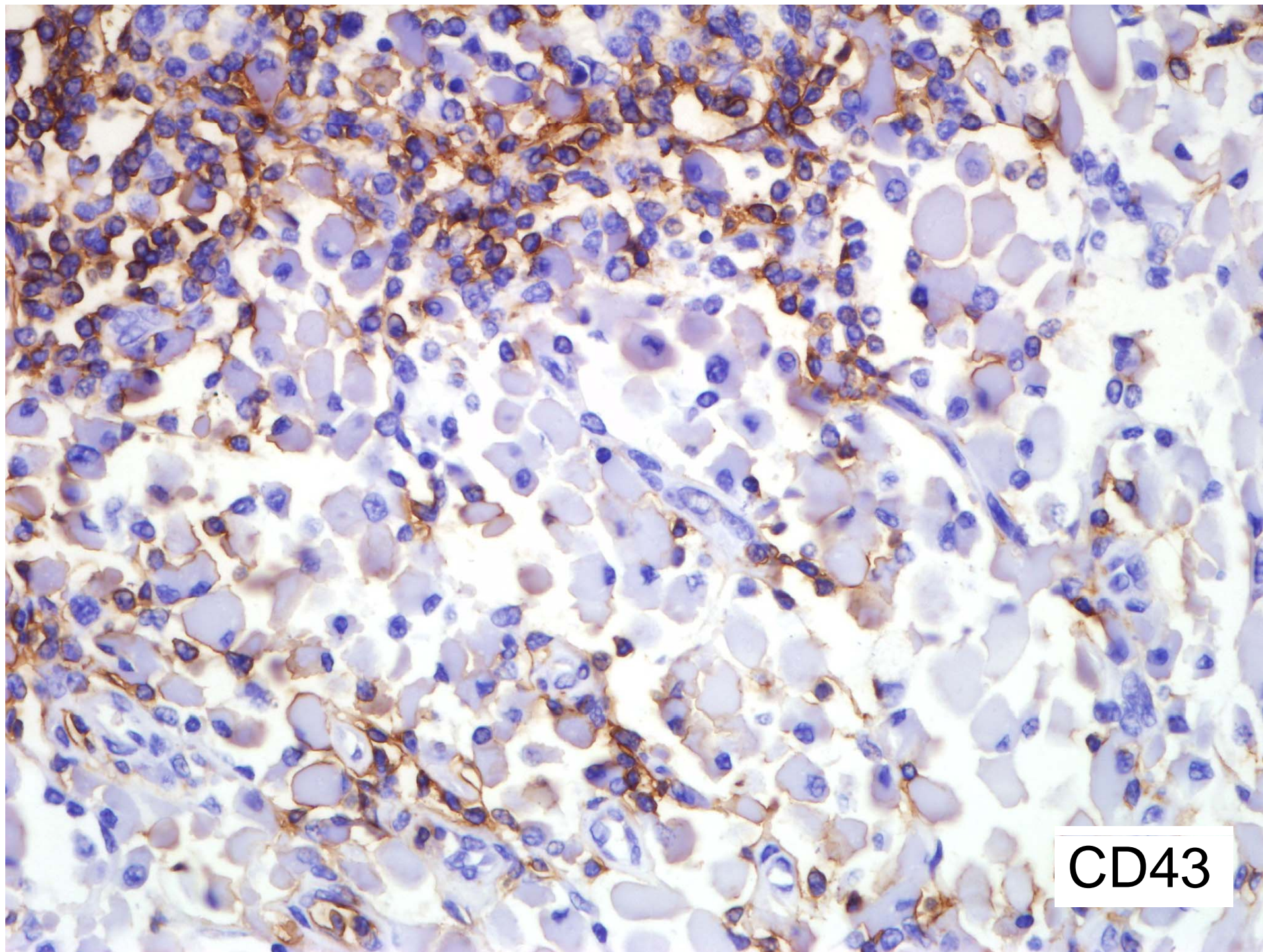

CD43

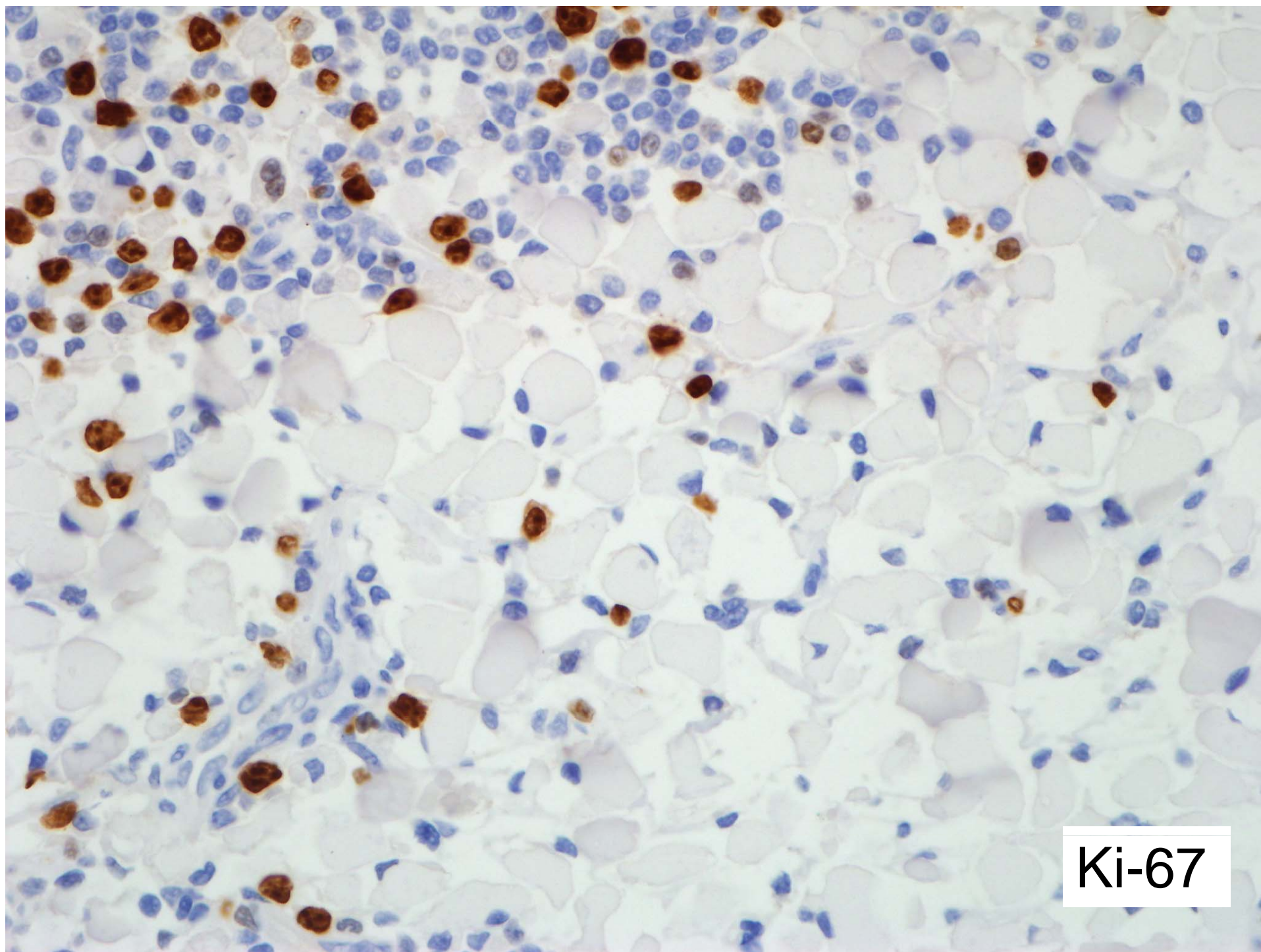

Ki-67

Supplement: Additional file 1 — Supplementary immunohistochemical results. Immunohistochemical detection of LCA, PAX-5, CD5, CD20, CD43 and Ki-67 (original magnification, ×400). [file 1752-1947-5-53-S1.PDF]
